# Brassicaceae Isothiocyanate-Mediated Alleviation of Soil-Borne Diseases

**DOI:** 10.3390/plants14081200

**Published:** 2025-04-12

**Authors:** Tikkisetty Pavana Praneetha, Sam A. Masih, Rosangela Addesso, Ann Maxton, Adriano Sofo

**Affiliations:** 1 Department of Genetics and Plant Breeding, Sam Higginbottom University of Agriculture, Technology and Sciences, Prayagraj 211007, India; pavanapraneetha@gmail.com; 2 Department of Molecular and Cellular Engineering, Sam Higginbottom University of Agriculture, Technology and Sciences, Prayagraj 211007, India; sam.masih@shiats.edu.in; 3 Department of Agricultural, Forestry, Food and Environmental Sciences (DAFE), University of Basilicata, 85100 Potenza, Italy; rosangela.addesso@unibas.it

**Keywords:** Brassicaceae, biofumigation, isothiocyanates, soil-borne pathogens, sustainable agriculture

## Abstract

Soil-borne diseases lead to high risk in crop production by diminishing the productivity and general health of the affected plants. *Brassica* plants are known to produce glucosinolates, which, upon decomposition, release bioactive isothiocyanates (ITCs). ITCs have attracted attention because of their biofumigation properties, effectively suppressing soil-borne pathogens and pests, promising natural solutions for managing soil-borne diseases. ITCs produced by *Brassica* plants or seed meal additives to soil have the ability to reduce soil-borne pests and diseases while increasing beneficial soil microbiota. Several researchers have indicated that ITCs can interfere with the life cycles of soil-borne pathogens and, at the same time, strengthen plant defense systems, which makes them a more environmentally friendly option than chemical pesticides. The breakdown of *Brassica* biomass has also been shown to stimulate beneficial microbial communities, which play a key role in nutrient availability and pathogen suppression. Studies indicate that this process enhances the availability of essential nutrients like sulfur and nitrogen in the soil, both of which are critical for plant growth and development. This review provides a comprehensive exploration of the role of *Brassica* ITCs in mitigating soil-borne diseases. We aim to consolidate current knowledge on ITC-mediated biofumigation, recommend strategies for enhancing its efficiency in practical applications, and highlight the need for future research to optimize its long-term effectiveness in sustainable agriculture.

## 1. Introduction

Soil-borne diseases pose significant challenges to agricultural productivity and sustainability worldwide [1]. Traditional methods of disease control often rely on chemical pesticides, which can have a negative impact on the environment and human health. To address these concerns, there is growing interest in alternative sustainable and environmentally friendly approaches. Among these methods, biofumigation, which involves the use of crops known for their ability to produce volatile bioactive compounds, has proven to be a promising strategy [1,2]. Biofumigation involves using plants’ natural defense systems to release biotoxic compounds into the soil atmosphere while organic amendments decompose. It includes green manure crops that produce biocidal molecules in the soil through the decomposition of freshly chopped plant material buried for this purpose. Among them, crops from the Brassicaceae family, like mustard and broccoli, have attracted attention for their capacity to inhibit soilborne pathogens through the release of isothiocyanates (ITCs) [1,2,3]. These compounds are derived from glucosinolates, which are abundant in brassicaceous species and are activated during tissue damage or breakdown [4]. Plants like mustard, cauliflower, and broccoli from the Brassicaceae family are commonly used due to their high glucosinolate levels. When these glucosinolates break down, they release toxic isothiocyanates that harm soil microorganisms [5,6]. However, this natural process is less harmful and longer-lasting than using synthetic fumigants. ITCs exhibit potent antimicrobial properties against a wide range of pathogens, making biofumigation-based crop rotation strategies and Brassicaceae-based crop rotations promising avenues for disease management in agriculture [1,4]. The concept of using brassicaceous crops to suppress disease is not new; historical agricultural practices have long recognized their benefits in crop rotation systems. However, recent scientific advances have deepened our understanding of the mechanisms by which *Brassica*-derived ITCs interact with soil microbiota and pathogens, providing new insights into their applications in modern agriculture [7].

To complement the disease-suppression benefits of *Brassica* crops, recent studies have highlighted their potential to improve soil health. As *Brassica* residues break down in the soil, they enrich the soil with organic matter, improving its fertility and structure. It has also been demonstrated that the breakdown of *Brassica* biomass activates beneficial microbial populations, which can enhance disease control and nutrient availability. Studies show that this procedure improves the soil’s availability of vital nutrients like nitrogen and sulfur, which are both necessary for plant growth and development [5]. This dual benefit of disease suppression and soil health improvement positions *Brassica* crops as a cornerstone of sustainable agricultural practices.

In this review, biochemical pathways involved in glucosinolate degradation and ITC release will be explored, along with the mechanisms by which ITCs exert their antimicrobial effects on soil ecosystems [8]. Practical applications, such as methods of biofumigation and strategies for integrated pest management, will also be discussed. Additionally, new research patterns and possible directions for future exploration, including improving the effectiveness and long-term viability of *Brassica*-based disease control methods, will be addressed.

Despite the growing interest in biofumigation, previous studies have often been fragmented, focusing either on specific aspects of *Brassica* isothiocyanates (ITCs) or their effects on a limited range of soil-borne pathogens, leaving gaps in understanding their broader antimicrobial potential and ecological impacts [8]. This review explores the role of *Brassica* ITCs in mitigating soil-borne diseases by consolidating existing knowledge and addressing these gaps. It delves into the biochemical pathways of glucosinolate degradation, the mechanisms through which ITCs exert antimicrobial effects, and their influence on the soil ecosystem. Furthermore, this review emphasizes practical applications, such as biofumigation methods and strategies for the inclusion of biofumigation in integrated pest management (IPM), through a combination of using cultural techniques, such as switching between crops, choosing pest-resistant cultivars, and planting pest-free rootstock with methods like biological control and habitat alteration. IPM is an ecosystem-based approach that aims to prevent pests or their damage over the long term. The review highlights the distinct advantages and long-term viability of natural plant-based solutions over synthetic fumigants, offering valuable insights for advancing sustainable agriculture.

## 2. Biofumigation Crops

Biofumigation is an organic approach to soil pest control that uses specific crops that have the potential to control soil-borne diseases and pests as a method to replace chemical fumigants such as methyl bromide, which negatively affect the environment and human health [9]. Some Brassicaceae species, including *Brassica juncea* and *Brassica carinata,* are potent biofumigants since they provide high glucosinolate levels that enhance the control of soil-borne diseases [9,10]. These species, together with *Sinapis alba* and *Raphanus sativus*, exhibit the status of the biomass yield and glucosinolate content that are useful for one or another kind of biofumigation, depending on the crop calendar and the climate [9]. In addition, biofumigation with *Sinapis alba* and *Raphanus sativus* improves the soil quality factors that support biological activities by reducing nematode loads [9,11].

Indian mustard (*B. juncea*), black mustard (*B. nigra*), white mustard (*Sinapis alba*), and yellow mustard (*B. hirta*) are used as biofumigation crops to control weeds and diseases that cause wilting [10] since they hold large quantities of glucosinolates within. Radish (*Raphanus sativus*) is known for its ability to suppress cyst nematodes in the soil. Nematode treatment is also carried out using rapeseed species like *B. napus* and *B. rapa*. *B. rapa* effectively controls bacterial wilt induced by *Ralstonia solanacearum* in tomatoes [12]. *Rhizoctonia solani*, a soil-borne pathogen with a wide host range, is dramatically decreased by degrading *B. juncea* plant tissues [13]. Volatile compounds from biofumigation crops suppress *Fusarium oxysporum, Rhizoctonia solani, Phytophthora nicotianae,* and *Verticillium dahlia* [14]. The use of green cover crops lowers *Verticillium* in potatoes; *Pythium, Fusarium*, and *Rhizoctonia* in beans; *Pythium* in lettuce; *Aphanomyces* rot in onions; *Pythium, Rhizoctonia*, and *Fusarium* root rot in peas; and *Fusarium* in carrot [15]. Besides being effective against pathogens, biofumigation has the potential to control weeds. Biofumigation with *B. juncea* crops can reduce weeds [16]. Incorporating oriental mustard (*B. juncea*) seed meal onto the field reduces weed growth [16], while allyl isothiocyanates can limit the growth of weeds, such as redroot pigweed [17]. Moreover, biofumigation with *B. juncea* has been studied for the control of non-native grasses in rangelands and seems to have the potential for efficient integrated weed management but requires more extensive field trials [18].

## 3. Biofumigation Techniques

**Figure 1:**
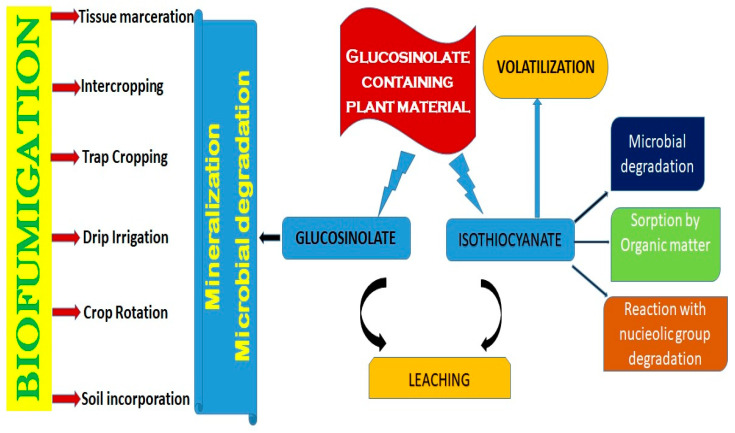
Biofumigation techniques used for mineralization of soil and degradation of soil pathogens.

Various techniques, viz., crop rotation, intercropping, and soil incorporation, are employed to harness the biocidal properties of plants (Figure 1). Additionally, combining biofumigation with other practices like solarization and plastic mulching can further enhance its efficacy. The following process could be followed for successful biofumigation.

First, soil incorporation should be performed in the morning or evening before the mustard crop has reached full bloom with a good moisture level. It is essential to use a flail mower to chop and crush as much plant material as possible before the actual integration. Since 80% of the fumigant gas is emitted in the first 20 min after mowing, the crop must be used right away. The field should then be rolled and packed to capture the fumigant gas in the soil. It is best to cover the area with tarps to trap the gas in the soil to improve the biofumigation effect. Then, after 14 days, the field should be left undisturbed to allow the plant material to decompose [1].

Intercropping is the practice of planting two or more crops in the same field. This technique can help suppress pests and diseases by creating a diverse ecosystem that is less hospitable to pests. Crop rotation is the process of planting different crops in the same field in a predetermined order over time. This approach can disrupt the life cycles of pests and pathogens, thus lowering their populations. Soil incorporation involves tilling plant material into the soil. This technique can release biocidal compounds from the plant material, suppressing pests and diseases. *Brassica* species, such as mustard and rapeseed, are commonly used for this purpose [19].

Solarization involves covering the soil with transparent plastic mulch to heat it up and kill pests and pathogens [19,20]. Combining soil incorporation with solarization can enhance the biocidal effect, as the heat can help to release biocidal compounds from the plant material more rapidly [20]. Trap farming entails growing a crop that draws pests away from the primary crop. When employed for biofumigation, many brassicaceous plants can also serve as trap crops, drawing in nematodes and other pests. Plant-parasitic nematodes, for instance, can be efficiently trapped by certain brassica plants that promote their hatching and activity without permitting their reproduction [19]. Incorporating these plants into the soil reduces nematode pressure in the field by fumigating the trapped worms. Once the pests are attracted to the trap crop, the plant material is macerated (crushed) and incorporated into the soil. Covering the soil with plastic mulch can create a microclimate that favors the release of biocidal compounds and further suppresses pests [20]. Tissue maceration involves crushing plant tissue to release biocidal compounds. The macerated plant material is subsequently integrated into the soil to prevent pests and diseases [20]. This technique can be used with a variety of plant materials, including *Brassica* species. Watering the soil after incorporating macerated plant material can help to activate and release biocidal compounds more effectively. This technique can be particularly useful in dry conditions [20]. Rolling the soil surface after incorporating macerated plant material can help to incorporate the plant material more deeply into the soil, enhancing the biocidal effect. This technique is often used in combination with other techniques, like solarization [20]. Like the trap cropping technique [21], covering the soil with a plastic mulch after incorporating macerated plant material can create a microclimate that favors the release of biocidal compounds and suppresses pests [20]. Drip irrigation can be used to apply liquid formulations of biocidal compounds directly to the soil. This technique can be more targeted than other methods, allowing for precise application of the biocidal agent [22].

## 4. Mechanism of Action

Glucosinolates and isothiocyanates play a crucial role in biofumigation with Brassicaceae species. The breakdown of glucosinolates into isothiocyanates upon tissue disruption creates bioactive compounds that target and inhibit the growth of multiple soil-borne pathogens and pests [2,5,6,23]. The effectiveness of biofumigation is dependent on the type and concentration of glucosinolates, which determine the release of potent isothiocyanates for effective pest control. Table 1 provides insight into the chemical structures of glucosinolates and isothiocyanates, as well as their roles in biofumigation and pest control.

### Mode of Action of Biofumigant Crops

The mode of action of biofumigant crops involves the inhibition of pathogen growth, enzyme activity, and cellular functions [24,25]. Suppression can be caused directly by biocidal toxicity or indirectly by alterations in the microbial community and soil fauna. After the use of biofumigant crops, it can be seen that the population of beneficial microorganisms, such as mycorrhizal fungi, increased [1]. Table 2 summarizes the biofumigation process and its mechanisms, the process of glucosinolate breakdown, and its pesticidal effects.

## 5. Role of Glucosinolates and Isothiocyanates in Biofumigation

High levels of glucosinolates (GSLs), which are stored in plant cells apart from the enzyme myrosinase, are produced by a variety of cruciferous species. When undamaged, GSLs do not pose a threat to microorganisms. However, myrosinase and GSLs interact when plant cells rupture, and myrosinase then hydrolyzes GSLs when water is present [23,24]. Isothiocyanates (ITCs) are among the several compounds produced by this process. GSLs are characterized as aliphatic, aromatic, or indole based on the kind of side chain (R), which affects the biological activity of the hydrolysis products [27].

The biological activity of the ITCs is influenced by the retention of the R group in the GSLs. Commonly utilized biofumigant plant species such as brown mustard, white mustard, radish, and rocket contain distinct GSLs, which contribute to the release of diverse ITCs (Table 3). Some biofumigants may have a combination of GSLs, while others may have a dominating GSL [28]. Even though ITCs are thought to be the most bioactive of the hydrolysis products and have typically been the subject of research pertaining to biofumigation, other substances, like fatty acids, nitriles, ionic thiocyanates, and non-glucosinolate sulfur-containing compounds, may also have an impact on pest and pathogen populations [17].

## 6. Myrosinase Activity Mediating Glucosinolate Breakdown

**Figure 2:**
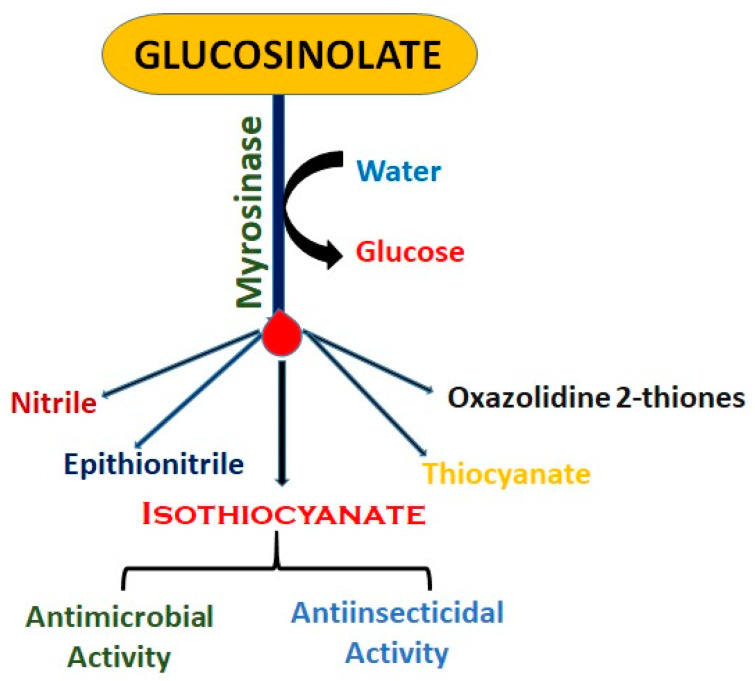
Products of glucosinolate breakdown by myrosinase.

Myrosinase is generally responsible for GSL hydrolysis. When these vegetables are sliced, chewed, or otherwise damaged, glucosinolates are broken down by myrosinase [23], and several bioactive compounds are released (Figure 2). This enzyme is activated when plant tissue is damaged (e.g., by chopping or chewing). The products depend on the glucosinolate structure and the conditions of the breakdown [34].

Besides being effective against microorganisms, GSL breakdown products have shown potential as anti-cancer agents by promoting the elimination of carcinogens, inducing apoptosis (programmed cell death) in cancerous cells, and inhibiting tumor growth.

Breakdown products include the following:

Among the various hydrolysates, isothiocyanates have attracted much attention because of their potent anti-cancer activity [23].

- Isothiocyanates: These are among the most commonly studied products of glucosinolate breakdown.
  - Sulforaphane: A secondary metabolite produced as a result of the hydrolysis of the glucosinolate glucoraphanin by myrosinase. It has been identified as the most effective active substance [35]. It is a potent anticancer phytochemical found in high levels in broccoli and provides cancer protection through the alteration of various epigenetic and non-epigenetic pathways. It is a safe-to-consume and potent anticancer phytochemical [36].
  - Phenethyl isothiocyanate (PEITC): Found in watercress, PEITC has been shown to inhibit the growth of cancer cells [31].
- Indoles: Another class of compounds formed from glucosinolate breakdown, such as indole-3-carbinol (I3C), which has been studied for its potential to modulate estrogen metabolism and reduce the risk of hormone-related cancers [31,37].
- Nitriles: The formation of nitriles can be favored in certain pH conditions or by the presence of specific proteins. Nitriles are less bioactive than isothiocyanates but can still play a role in the plant’s defense mechanisms [23]
- Thiocyanates: These compounds can affect thyroid function by competing with iodine uptake, potentially impacting thyroid hormone production. However, the effect is generally considered minor in the context of a balanced diet [38].

## 7. Isothiocyanate Breakdown Mechanism

The hydrolysis reaction catalyzed by myrosinase leads to the formation of ITCs, which are highly reactive and can exhibit potent pesticidal and antimicrobial properties. Isothiocyanates, such as allyl ITC, are considered the main bioactive compounds produced during this process. ITCs are known to disrupt the cell membranes of pathogens, including fungi [24], bacteria [39], and nematodes [40], thereby inhibiting their growth. Different glucosinolates (e.g., sinigrin, sinalbin) yield different ITCs with varying structures and biological activities [29]. While ITCs have significant biocidal activity, the other products of breakdown can also contribute to the plant’s defense mechanisms.

### Breakdown of GSL

When *Brassica* plants are damaged, including chopping or crushing, myrosinase meets the stored glucosinolates within the plant cells [6]. This interaction leads to the breakdown of glucosinolates into various byproducts. Allyl isothiocyanate (AITC) is one of the most commonly studied and bioactive isothiocyanates and is released when the glucosinolate sinigrin undergoes hydrolysis [29,30]. Allyl ITC is highly toxic to a variety of soilborne pathogens, including bacteria, fungi, and nematodes, making it an effective tool for biofumigation. Additionally, the breakdown of sinigrin into allyl isothiocyanate is influenced by factors like pH, moisture, and temperature, which can optimize its pesticidal and antimicrobial effects [41].

## 8. Maximizing ITC-Mediated Disease Suppression

The ways in which biofumigation can be optimized can be summarized as follows:

- Different biofumigant crops must be tested for activity against the target pathogen. This can be achieved through in vitro studies, particularly focusing on the effect on resting structures such as chlamydospores, sclerotia, and microsclerotia, or ideally in soil-based assays under controlled conditions to establish the biofumigant with the best potential for a specific soilborne disease before extensive field experiments are performed. Recently, an optical platform was devised [42] using cation-exchanging Nafion particles to produce transparent soil to study the mechanisms of transmission of pathogens on lettuce plants. This platform may be utilized as a real-time biological screen to examine the effects on target pathogens, as it was shown that transparent soil is ideal for imaging studies of certain plant–microbe interactions in situ, as soil microbes and their interactions with plants enhance the supply of nutrients via nodulation or biological fertilization.
- *Brassica* species producing aliphatic short-chain ITCs may be more efficient than those producing long-chain aromatic ITCs due to enhanced volatility and lower sorption of these chemicals to organic debris. The biofumigant species may also need to be chosen based on winter hardiness, growth rate, and GSL production during various times of the year, depending on when they are to be integrated. Seed meals and processed biofumigants may be more appropriate (1) for small, intensively cropped areas such as greenhouses and polytunnels and (2) for the suppression of more resistant resting structures, such as *Verticillium dahliae*’s microsclerotia [43].
- Agronomic aspects, including the seed rate, sowing time, fertilizer treatment, and the best time for inclusion, must all be taken into account in order to maximize the production of biofumigant crops and the GSL level since biofumigation requires large volumes of biomass. For example, fertilization-mediated nitrogen and sulfur delivery has been shown to alter the amount of GSLs in plant tissue [44].
- The breakdown of *Brassica* plant cells is essential for converting glucosinolates into isothiocyanates (ITCs). The release of ITCs is proportional to the degree of cell damage, with a higher level of disruption leading to increased ITC concentrations [45]. Therefore, pulverizing and crushing plant material is a better use of equipment than chopping. Finely shredding the biofumigant crop before incorporation into the soil is crucial for maximizing ITC release [46].

Biofumigation is compatible with sustainable farming systems and works well in combination with other biological control methods. Successful integration of biofumigation into agroecosystems requires careful planning and strategic crop rotation. Strategies include pairing biofumigation with cover cropping, adopting organic farming techniques, and applying precision agriculture methods to maximize its benefits [15].

In order to maximize GSL hydrolysis, immediate inclusion is necessary, along with the addition of water. Apart from this, tarping or soil sealing will maximize ITC retention.

The use of biofumigant crops in green manures, seed meals, and plant residues highlights their versatility in agricultural pest management (Table 4). *Brassica*-based biofumigation has demonstrated effectiveness against diverse pathogens, including *Rhizoctonia solani, Phytophthora nicotianae*, and *Fusarium spp*., as well as soilborne diseases like scab and damping off [47]. Its adaptability across different crops and pest challenges ensures broad applications in integrated pest management systems.

## 9. Effectiveness of Processed Biogumifationts Against Pathogens

Processed biofumigants are advantageous since they can be used when biofumigant crop cultivation is impractical, such as during the winter [61]. They integrate easily into crop rotations and are particularly suitable for intensive agricultural systems. *Brassica* seed meals, particularly from mustard crops, are well-suited for soil amendments. These seeds are rich in glucosinolate (GSL) content and retain the myrosinase enzyme necessary for converting glucosinolates into isothiocyanates (ITCs) that can suppress soil-borne pests and diseases. Research confirms their effectiveness in controlling soil-borne microbial diseases [1]. Researchers have shown efficacy against pathogens like *Rhizoctonia* spp. Mustard (*B. juncea*) releases isothiocyanate, which suppresses the activity of *Pythium* spp. when mustard seed meal is incorporated into the soil [62]. Studies on ground seeds from three *Brassica* species identified allyl isothiocyanate as a fungicidal compound that controls *Rhizoctonia* damping-off in cabbage plants. A liquid formulation derived from defatted *B. carinata* seed meal effectively reduces the activity of the root-knot nematode *Meloidogyne incognita* [63].

### Effectiveness Against Pathogens

1. Factors Affecting Efficacy: The following variables determine the success of biofumigation: soil conditions, climate, and farm management practices, as soil with low organic matter is favorable for biofumigation, and factors like pH, temperature, soil-metal interaction, and water management in farms influence the release of bioactive compounds [11,44,64].
2. Knowledge Gaps and Limitations: Research on the impact of biofumigation on free-living nematode species remains limited. A major challenge in commercial adoption is the lack of a consistent experimental framework, leading to variable control outcomes across organisms and studies, as the effects of ITCs on fungal activities include the inhibition of growth and germination of sclerotia or spores [24]. Additional research is required to identify the most effective biofumigants for specific pathogens under diverse conditions.
3. Significance of effects on soil health: Biofumigation enhances soil health by promoting greater microbial diversity and activity within the soil ecosystem [65]. The addition of *Brassica* residues improves the soil structure by enhancing organic matter, thus improving nutrient availability. These benefits contribute to increased soil fertility and resilience to environmental stresses, fostering a sustainable farming system [66,67].

## 10. Inherent Limitations of Biofumigation and Challenges

Despite its numerous advantages, biofumigation faces several challenges that can limit its widespread adoption and effectiveness.

- Pathogen-Specific Limitations: The concentration of ITCs needed to suppress pathogens varies depending on the target soil-borne pathogens, nematodes, or weed seeds [16,68]. For example, for *Verticillium dahliae*, *Brassica* plants may not produce enough ITCs to control its resilient microsclerotia under field conditions [43].
- Soil-Dependent Limitations: The type of soil significantly influences biofumigation outcomes. Lighter soils with sandy textures and a low organic matter content are more favorable for biofumigation [64]. High organic matter in soil reduces ITC availability due to sorption, diminishing their effectiveness against pathogens [69]. In contrast, sandy soils enable better diffusion of toxic gases, enhancing biofumigation efficacy. Variability in glucosinolate levels: The effectiveness of biofumigation relies heavily on the type and concentration of glucosinolates (GSLs) in the *Brassica* species used. GSL levels can vary significantly between species, cultivars, and even different parts of the plant, making it difficult to standardize its application for consistent results [70].
- Potential Harm to Future Crops: The residual effects of biofumigation, such as an excessive release of isothiocyanates (ITCs), may cause phytotoxicity to subsequent crops if not managed properly. Sensitive crops planted immediately after biofumigation can suffer damage, which necessitates careful planning of crop rotation [9].
- Timing and Application Techniques: The success of biofumigation depends on precise timing and application methods [9]. Factors such as the plant growth stage, pH, incorporation into soil, water management, and moisture levels significantly influence the release and effectiveness of bioactive compounds. Incorrect timing or poor technique may result in reduced efficacy or inconsistent pest and disease control [9,71,72].
- Environmental Influences: Soil conditions, temperature, and microbial activity can alter the hydrolysis of glucosinolates [6] and the release of ITCs. Adverse environmental factors, including local environmental conditions, may limit the biofumigation process or reduce its effectiveness in pest suppression [73]. Several studies have detected both GSLs and ITCs in the rhizosphere, which have been implicated in the suppression of pests and pathogens [74].
- Economic Feasibility: The cost of cultivating biofumigant crops into existing farming systems can be prohibitive for small-scale farmers. This challenge is further compounded by the lack of access to optimized cultivars for specific biofumigation purposes.
- Regulatory and Adoption Barriers: The adoption of biofumigation practices may be hindered by limited awareness, a lack of extension services, and the need for regulatory frameworks to ensure the safety and sustainability of the practice.

## 11. Conclusions and Future Scope

*Brassica* isothiocyanate (ITC)-mediated biofumigation represents a powerful and sustainable strategy for managing soil-borne diseases in agricultural systems. The decomposition of glucosinolates in *Brassica* plants, resulting in the release of ITCs, offers a natural and effective means of suppressing a broad spectrum of soil-borne pathogens, including fungi, nematodes, and other pests. The antimicrobial efficacy of ITCs is shaped by their chemical structure, with aromatic and aliphatic ITCs exhibiting distinct toxicological profiles and pathogen-specific effects. This versatility underscores their potential in tailored pest management approaches. Research has highlighted that ITCs not only reduce pathogen populations but also contribute to improved soil health by influencing microbial community dynamics. The dual role of ITCs in pathogen suppression and soil health enhancement makes them a cornerstone of advancing agroecological practices. Furthermore, the practical application of *Brassica* residues or seed meal amendments has demonstrated significant promise in field settings, though optimization of these methods is necessary for greater efficacy and scalability. Biofumigation represents a promising strategy for sustainable plant disease management in agroecological systems. Its ability to control pathogens while improving soil health underscores its potential to support resilient and environmentally friendly agricultural practices. Continued research and adoption of biofumigation will contribute to advancing sustainable agriculture globally. Future exploration should focus on further elucidating the biochemical pathways of glucosinolate degradation, improving ITC release mechanisms, and understanding the long-term ecological impacts of *Brassica*-mediated biofumigation. Additionally, integrating this approach with other sustainable agricultural practices can enhance its effectiveness and contribute to holistic pest and disease management strategies. In conclusion, *Brassica* ITC-mediated biofumigation provides a resilient and environmentally friendly way to control soil-borne diseases, supporting the global transition toward sustainable agriculture. With continued research and thoughtful implementation, this approach holds significant potential to mitigate crop losses, enhance soil health, and promote agricultural resilience in the face of evolving environmental challenges. Additionally, long-term studies are crucial to assess biofumigation’s impact on soil health, microbial diversity, and nutrient cycling to ensure environmental sustainability. Investigating its role in promoting biodiversity, improving pest suppression in various agroecological settings, and supporting crop productivity over time can provide insights for integrating biofumigation into resilient farming systems. Expanding collaborations between researchers, farmers, and policymakers will also be key in overcoming adoption barriers and scaling this technique effectively.

## Figures and Tables

**Table 1:** Chemical structures and role of glucosinolates and isothiocyanates.

Compound/Structure	Description
**Glucosinolate** 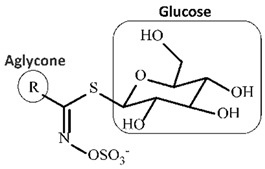	Glucosinolates are naturally occurring sulfur-containing chemicals, principally found in plants from the Brassicaceae family, such as mustard, broccoli, and cabbage. The structure consists of a glucose molecule (C_6_H_11_O_5_) attached to a sulfur-containing group (SO_3_) and a variable side chain (R), which is responsible for the compound’s specific properties. When the plant tissue is disrupted, either by mechanical damage or decomposition, glucosinolates break down enzymatically into biologically active products, primarily isothiocyanates, through the action of the enzyme myrosinase. The breakdown of glucosinolates into isothiocyanates and other products like thiocyanates, nitriles, and epithionitriles contributes to the plant’s defense mechanism against herbivores, pests, and pathogens [6].
**Isothiocyanate** 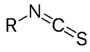	Isothiocyanates are the principal active products released from the breakdown of glucosinolates. The chemical structure consists of a nitrogen atom (N) double-bonded to a carbon (C) atom, which is also double-bonded to a sulfur (S) atom. The side chain (R) of the isothiocyanate molecule is derived from the original glucosinolate and varies depending on the specific *Brassica* species. These compounds have potent pesticidal properties and are known for their ability to disrupt the cellular processes of a wide range of pathogens, including fungi, bacteria, and nematodes. The presence of the isothiocyanate group (-N=C=S) is crucial for its antimicrobial activity. Isothiocyanates inhibit pathogen growth by affecting the enzymes involved in cellular metabolism and can act as effective biocides when released into the soil. The specificity and effectiveness of these compounds depend on the type and concentration of glucosinolates in the plant tissue, as well as the conditions under which they are released [2,23].

**Table 2:** Biofumigation process and its mechanisms.

Step	Description	Detailed Explanation with Citations
Tissue Disruption	Tissue damage or decomposition of biofumigant crops releases bioactive compounds.	When biofumigant crops like *Brassica* species (e.g., mustard, rapeseed) are damaged, either mechanically (through crushing or tilling) or through natural decomposition, the plant cells release glucosinolates, which, when broken down, produce various biologically active compounds, primarily isothiocyanates, and other breakdown products like nitriles and thiocyanates [25].
Release of Isothiocyanates	Glucosinolates break down into isothiocyanates due to enzymatic action by myrosinase.	The breakdown of glucosinolates occurs via an enzyme called myrosinase, which is released when plant tissues are damaged. Myrosinase catalyzes the conversion of glucosinolates into isothiocyanates. The type of isothiocyanate produced depends on the side chain of the original glucosinolate [25].
Inhibition of Pathogen Growth	Isothiocyanates, along with other breakdown products, interrupt the cellular processes of pathogens.	Isothiocyanates inhibit pathogen growth by disrupting various cellular processes. They can interfere with enzyme function, protein synthesis, and the integrity of the pathogen’s cellular membrane. For example, isothiocyanates bind to proteins and enzymes in microorganisms or pests, which inhibits their metabolic processes, leading to their death. These compounds can also disrupt fungal cell membranes, making them highly effective in controlling soil-borne diseases [24].
Targeted Pest Control	The specificity of isothiocyanates provides targeted control of pests, pathogens, and nematodes without harming beneficial organisms.	The specificity and efficacy of biofumigants depend on the concentration and type of glucosinolates in the crop, which determines the type and potency of the isothiocyanates released. While these compounds are extremely toxic to pests and pathogens, they have minimal negative impact on beneficial organisms such as earthworms and plant roots, ensuring sustainable pest control [26].
Reduction in Synthetic Chemical Use	The natural release of bioactive compounds reduces the need for synthetic fumigants or pesticides.	By using biofumigant crops, farmers can reduce or eliminate the need for synthetic chemical pesticides, which can have negative environmental and health consequences. Biofumigation with plants like *Brassica* species provides a more environmentally friendly, sustainable approach to pest control while maintaining soil health. Additionally, the practice can improve soil quality over time by increasing organic matter content through crop decomposition [24]

**Table 3:** Some commonly used biofumigant crops and their respective GSLs and ITCs.

Common Name	GSL (Glucosinolate)	ITC (Isothiocyanate)	Structure	Reference
Brown mustard (*Brassica juncea*)	Sinigrin	2-propenyl-ITC (= allyl-ITC)	CH_2_=CH-CH_2_-N=C=S	[29]
Black mustard (*Brassica nigra*)	Sinigrin	2-propenyl-ITC (= allyl-ITC)	CH_2_=CH-CH_2_-N=C=S	[30]
White mustard (*Sinapis alba*)	Sinalbin	4-hydroxybenzyl-ITC	HO-C_6_H_4_-CH_2_-N=C=S (benzene ring)	[29]
Radish (*Raphanus sativus*)	Glucoraphenin	4-methylsulfinyl-3-butenyl-ITC	CH_3_-SO-(CH_2_)-CH=CH-N=C=S	[31]
Rocket (*Eruca sativa*)	Glucoerucin	4-methylthiobutyl-ITC	CH_3_-S-(CH_2_)_3_-N=C=S	[31]
Cabbage (*Brassica oleracea*)	Glucobrassicin	3-indolylmethyl-ITC	C_6_H_4_-CH_2_-N=C=S	[32]
Broccoli (*Brassica oleracea* var. italica)	Glucoraphanin	4-methylsulfinyl-3-butenyl-ITC	CH_3_-SO-(CH_2_)-CH=CH-N=C=S	[33]

**Table 4:** Examples of pathogens suppressed through biofumigation.

Biofumigant Crops/Method of Application	Name of Plant Disease /Pest	Causal Agent	References
*Brassica* residues	Common scab disease of potato	*Streptomyces scabies*	[48]
*B. juncea* as cover crop	Root rot of pea	*Aphanomyces euteiches*	[49]
*B. juncea* leaf extracts and green manures	White potato cyst nematode	*Globodera pallida*	[50]
*Brassica juncea* as seed meal, seed powder, dry and fresh plants, and methanol extract	Damping off of vegetables	*Rhizoctonia solani*	[13]
*B. napus* as seed meal	Apple root rot	*Rhizoctonia solani*	[51]
Mustard as cover crop	Lettuce drop	*Sclerotinia minor*	[52]
*B. juncea* and *B. napus* residues	All diseases of wheat	*Gaeumannomyces graminis* var. *tritici*	[53]
*B. oleracea* residues	Damping off diseases in greenhouses	*Pythium aphanidermatum*	[54]
*B. juncea* as seed meal	Soil-borne pathogenic fungi of soyabean	*Fusarium oxysporum, R. solani, Macrophomina phaseolina, Sclerotium rolfsii*	[55]
*B.carinata* as seed meal	Sugar beet damping off	*Pythium ultimum*	[56]
*B. oleracea* residues	Cabbage yellows	*F. oxysporum f.sp. conglutinans*	[57]
*Brassica* as cover crop	Woody ornamentals	*R. solani* and *Phytophthora*	[14]
*B.oleracea nicotianae, B. napus* residues	Wilt disease in cauliflower	*Verticillium dahliae*	[58]
*Brassica* spp. As green manure	Soil-borne diseases of potato	*Rhizoctonia solani, Phytophthora erythroseptica, Pythium ultimum, Sclerotinia sclerotiorum, and Fusarium sambucinam*	[59]
*B. napus* as green manure	Root-knot nematode on potato	*Meloidogyne chitwoodi*	[60]
*B. napus* as seed meals	Apple replant disease	*Phytophthora, Pythium* and *Rhizoctonia*	[61]

## Data Availability

All the data are included in the manuscript.
